# Co‐culturing or conditioned medium of Sertoli cells: Which one supports in vitro maturation of bovine oocytes and developmental competency of resulting embryos?

**DOI:** 10.1002/vms3.939

**Published:** 2022-09-09

**Authors:** Sina Nazifi, Hassan Nazari, Hossein Hassanpour, Ebrahim Ahmadi, Azita Afzali

**Affiliations:** ^1^ DVM Graduate Student, Faculty of Veterinary Medicine Shahrekord University Shahrekord Iran; ^2^ Research Institute of Animal Embryo Technology Shahrekord University Shahrekord Iran; ^3^ Department of Basic Sciences Faculty of Veterinary Medicine, Shahrekord University Shahrekord Iran; ^4^ Clinical Embryologist Shahrekord University of Medical Sciences Shahrekord Iran

**Keywords:** co‐culture, conditioned medium, embryo, oocyte in vitro maturation, Sertoli cell

## Abstract

**Background:**

Sertoli cells (SCs) as supportive cells in the seminiferous tubule play an essential role in the nutrition and development of adjacent cells by secreting several beneficial growth factors, stimulators and cytokines which can be conceived to improve the developmental competency of oocyte or embryo in the co‐culture system.

**Objectives:**

This study aimed to improve the maturation of bovine oocytes and consequently the development of resulting embryos in co‐culture with SCs and their conditioned medium (CM).

**Methods:**

The retrieved cumulus‐oocyte complexes (COCs) from the abattoir‐derived ovaries were matured in maturation medium alone (control group), in co‐culture with ovine SCs (co‐culture group), and in presence of 10% CM prepared in 33°C and 39°C (CM33 and CM39 groups). The nuclear maturation competency and subsequent embryo development rate of cultured COCs in all groups were evaluated.

**Results:**

The results of this study showed that SCs and CM increased meiosis resumption from GV to the MII compared to the control group, significantly (*p* < 0.05). Besides, the degenerated oocytes in the co‐culture group were significantly higher than those in the control, CM33 and CM39 groups (*p* < 0.05), and the lowest cleavage rate belonged to the co‐culture group (*p* < 0.05). The blastocyst rate was also lower in the co‐culture group than other groups and there was a significant difference between the control and two CM groups (*p* < 0.05).

**Conclusions:**

The Sertoli cells can be suitable for co‐culturing with oocytes during IVM but detrimental for subsequent embryo development. In turn, Sertoli cell‐derived conditioned medium (SC‐CM) can provide sufficient bioactive materials for COCs to enhancing oocyte competence and embryo development.

## Graphical abstract

Sertoli cell (SCs) can be suitable for co‐culturing with oocytes during IVM, but detrimental for subsequent embryo development. In turn, Sertoli cell‐derived conditioned medium (SC‐CM) can provide sufficient bioactive materials for COCs to enhancing oocyte competence and embryo development.

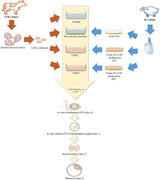

## 1 INTRODUCTION

In vitro embryo production is an interesting technique that has been used routinely in many mammalian species to increase the efficiency of reproduction without compromising its production life. Obtained many advances, besides several benefits of this approach have convinced the researchers to strive to increase its efficiencies. Nevertheless, recent studies have shown that the rate of pregnancy following the transfer of in vitro produced embryos is lower compared to in vivo counterparts (Banwell & Thompson, 2008).

The quality of the oocyte plays an important role in the successful production of embryo. The oocytes competency for maturation, growth, and nuclear and cytoplasmic development can be influenced by in vivo and in vitro factors including hormones and growth factors (Ali & Sirard, 2002). In particular, it has been suggested that one desirable approach for the improvement of in vitro maturation (IVM) condition is the application of feeder cells in terms of co‐culture systems to obtain higher yields of better quality oocytes (Feng et al., 2013; Lee et al., 2018). Presumably, the advantages of co‐culture systems are due to secreting bioactive materials such as growth factors, stimulators and cytokines and eliminating detrimental components, and decreasing oxidative stress in the co‐culturing microenvironment (Joo et al., 2001). Immature oocytes can be influenced by these factors to increase nuclear and cytoplasmic maturation and this can be used to improve the subsequent embryo development.

Sertoli cells (SCs) are one of the main support cells in the seminiferous tubule. These cells play an essential role in the nutrition and development of germ cells and subsequent spermatogenesis (Griswold, 1998). Undoubtedly, the positive effect of SCs is due to their secreted factors, which can improve the growth and developmental competency of other adjacent cells. SC‐secreted factors can be categorised as nutritional, environmental and regulatory factors (Skinner, 2005). Regulatory factors are defined as factors that affect cell function, growth or differentiation at the molecular level through receptor‐mediated signal transduction. These factors can act as endocrine factors to affect SCs or paracrine factors to affect adjacent cells (Skinner, 2005). The two main subsets of regulatory factors are growth factors and hormones. Growth factors, in addition to having a direct effect on the cell cycle, can also affect other types of cellular functions and cell differentiation. These growth factors that are secreted by SCs are including insulin growth factor I (IGF‐I), IGF‐II, epidermal growth factor (EGF), transforming growth factor α (TGFα), TGFβ, fibroblast growth factor (FGF), neurotrophins, interleukin‐1 (IL‐1), Kit ligand, stem cell factor (SCF) and glial cell line‐derived neurotrophic factor (GDNF) (Skinner, 2005). Unlike growth factors, hormones do not directly affect cell proliferation; rather, by stimulating the production of growth factors. The most important growth hormones secreted by SCs are activin/inhibin, anti‐mullerian hormone (AMH) and oestrogen.

Among the growth factors secreted by SCs, most of them have been used in the process of in vitro maturation of oocytes, such as IGF‐I (Purohit et al., 2005; Quetglas et al., 2001), EGF (Das et al., 1992; De La Fuente et al., 1999; Oyamada et al., 2004), FGF (Imani et al., 2007) and GDNF (Cui et al., 2018; Wang et al., 2018) which have often had positive effects on oocyte maturation and subsequent embryonic development. Many studies can be found on other growth factors which confirm their positive effects on oocyte maturation and embryo development.

Besides, the hormones secreted by SCs have been also used in the maturation medium of the oocyte. It has been shown that activin increases the oocyte maturation (Alak et al., 1998; Pang & Ge, 1999) and increases the subsequent produced blastocysts (Silva & Knight, 1998). AMH has also improved oocyte maturation in mice by increasing the expression of growth differentiation factor 9 (GDF9) and bone morphogenetic protein 15 (BMP15) factors (Zhang et al., 2014). Inhibin has been controversial effects and in most cases has an inhibitory role on oocyte maturation (Alak et al., 1996; van de Wiel et al., 1983); hence, due to inhibitory actions of LH (Luteinising Hormone) on inhibin secretion with SCs, in this study the hCG (Human Chorionic Gonadotropin) with high conserve homology and similar function with LH (Choi & Smitz, 2014) was used in the culture medium of SCs and IVM to suppression of inhibin secretion.

As feeder cells indirectly influence oocyte maturation through the transwell system (Lee, 2020, 2021), in this study, it was hypothesised that the SC‐derived conditioned medium (SC‐CM) might show similar or improved effects on oocyte development. The secreted factors that exist in the culture medium where the somatic cells are cultured, are referred to as secretome, microvesicle or exosome; therefore, the medium is named conditioned medium (CM). In this regard, the disadvantages of SCs co‐culture would be overcome, and there will be a possibility to highlight the potential function of SC‐CM as a paradigm to establish a reliable system for ARTs and give a new insight in reproduction field.

On the other hand, in this study, the SCs of ovine were used to evaluate its effect on bovine oocyte maturation and subsequent development of resulting embryos, due to the prevention of probable transmission of the routine infectious agents in bovine such as Infectious Bovine Rhinotracheitis (IBR) and Bovine Virus Diarrhoea (BVD), and production of bovine specific pathogen‐free embryos as a way to control transmission of diseases via feeder cells.

Due to the secretion of various IVM‐benefit growth factors, the Sertoli cells can lead to better results in oocyte maturation and subsequent embryo development than other differentiated somatic cells. Therefore, the present study aimed to investigate the improvement maturation of oocytes and consequently, the development of resulting embryos in co‐culture with Sertoli cells and its conditioned medium.

## 2 MATERIALS AND METHODS

### 2.1 Isolation, culture, and identification of ovine Sertoli cells

The testes of 1‐year‐old male sheep were collected from a local industrial slaughterhouse and transferred to the laboratory on frozen ice pack at 10–15°C. After washing and sterilise with 70% ethanol, the testes were decapsulated and minced into small pieces. The enzymatic digestion for Sertoli cell isolation was performed according to Hassanpour et al. (2015) with some modifications (Hassanpour et al., 2015). The minced fragments were washed with Dulbecco's Modified Eagle Medium‐Low glucose (DMEM‐LG; Gibco 31600‐083) and then resuspended in the first enzymatic digestion solution including DMEM‐LG containing 1 mg/ml collagenase IV (Gibco 17104‐019), 1 mg/ml hyaluronidase (Sigma‐Aldrich H2126), 1 mg/ml trypsin (Sigma‐Aldrich T6567) and 50 IU/ml DNase I (Sinaclon MO5401) for 30 min incubation in 39°C with horizontal shaking. The sample was then centrifuged in 30 × *g* for 30 s and the resulting precipitate was incubated with the second enzymatic digestion solution including DMEM‐LG containing 1 mg/ml collagenase IV, 1 mg/ml hyaluronidase and 50 IU/ml DNase I in 39°C for 45 min with horizontal shaking. After centrifuging the sample at 30 × *g* for 1 min, the supernatant was filtered through 70‐µm nylon mesh (BD Falcon). Finally, the obtained cells were washed twice and cultured in DMEM‐LG containing 20% foetal bovine serum (FBS; Gibco 10270) in 5% CO_2_ at either 33°C or 39°C. After 24 h, the suspended cells were removed. At this time the round to cuboidal cells that adhere to the Petri dish are SCs. The culture process is continued by refreshing the culture medium. At the second passage, the SCs were identified by immunocytochemistry through vimentin detection in cultured cells. In Brief, the 1 × 10^4^ cells were cultured on coverslips in each well of 12‐well cell culture dishes. After 48 h the cultured cells were washed with pre‐warmed medium and dried for 15 min. The dried cells were fixed in 4% paraformaldehyde for 10 min and permeabilised in 0.2% Triton X‐100 (Gibco 1.11869) in PBS for 15 min. The cells were washed twice with PBS containing 0.1% Tween 20 (Sigma‐Aldrich P1379) and 1% bovine serum albumin (PBST/BSA), and blocked with 5% sheep serum in TBS for 10 min and anti‐vimentin (Abcam, Cambridge, UK; 2 µg/ml PBST/BSA) was applied for 60 min at room temperature. After washing again, fluorescein isothiocyanate (FITC)‐conjugated sheep anti‐mouse Ig was diluted in PBST/BSA at a ratio of 1:50 and incubation was further continued for 45 min at room temperature. After washing with PBST/BSA, the nuclei were counterstained by Hoechst 33342 (Sigma‐Aldrich B2261) at 5 µg/ml for 5 min, then washed twice. The coverslips were then transferred and mounted onto microscope slides and examined under a fluorescence microscope (Olympus, Tokyo, Japan).

### 2.2 Preparation of conditioned medium (CM) of Sertoli cells

The Sertoli cells at the second passages were cultured in DMEM‐LG medium containing 10% FBS in 5% CO_2_ at either 33°C or 39°C until 80%–90% of confluency. After washing the cells twice with PBS to remove any residual factors and serum, the cells were re‐fed with serum‐free DMEM‐LG supplemented with 0.05 IU/ml FSH and 2 mM hCG for 72 h. Then the CM was collected and centrifuged twice, first at 1000 × *g* for 5 min at 4^°^C, then 4000 × *g* for 15 min at 14^°^C. The CM was finally filtrated through a 0.22‐µm filter to remove cellular debris, aliquoted and stored frozen at –30°C until use.

### 2.3 Preparation of SCs monolayer for co‐culture

The second passage of SCs was also used for co‐culture with immature bovine COCs. The SCs were cultured at 1 × 10^4^ cell/ml in 50 µl droplets of bicarbonate tissue culture medium‐199 (b‐TCM; Sigma‐Aldrich M5017) + 10% FBS at 39°C and 9% CO_2_ with maximum humidity under paraffin oil. Two hours before use, the culture medium was replaced by maturation medium (b‐TCM containing 10% FBS, 0.05 IU/ml FSH and 2 mM hCG).

### 2.4 In vitro maturation of bovine oocytes

Bovine ovaries were collected from a local slaughterhouse, transported to the laboratory in sterile normal saline solution within 2–3 h, and prepared for oocyte retrieval. All visible ovarian follicles with a diameter of 2–8 mm were aspirated using a 10 ml syringe fitted with an 18 g needle. The COCs with at least 3 layers of cumulus cells and uniform granulated cytoplasm with homogenous distribution of lipid droplets were selected and washed three times with HEPES‐TCM supplemented with 10% FBS. The selected oocytes were cultured in maturation medium at 39°C in 9% CO2 for 22–24 h.

### 2.5 Experimental design

**FIGURE 1:**
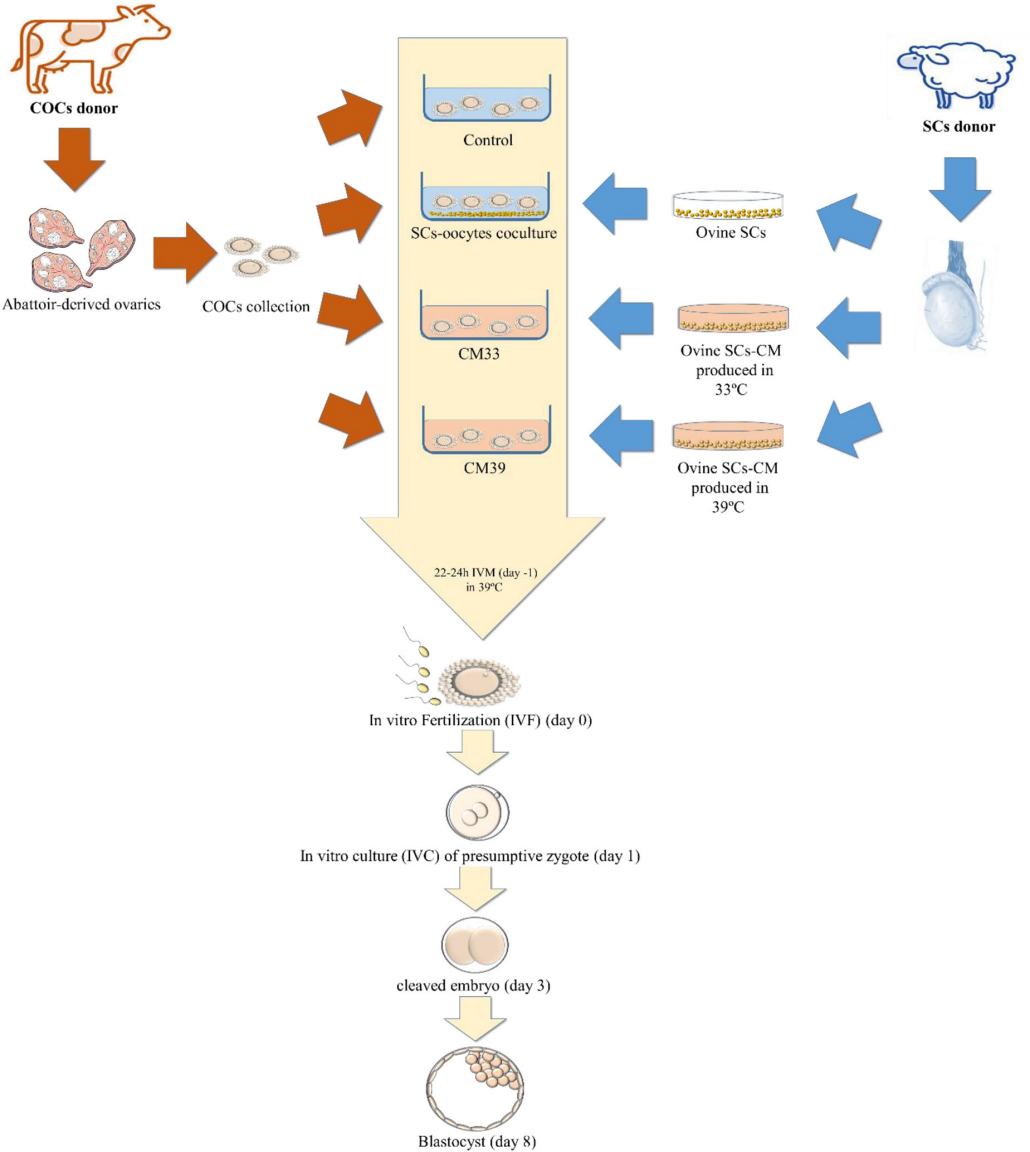
A schematic illustration of the experiments. Ovine Sertoli cells (SCs) co‐culture or supplementation of SC‐conditioned medium (CM) was applied to the bovine in vitro maturation (IVM). The maturation rate, oocyte degeneration rate, cleavage rate, and blastocyst rate were assessed at days –1, 1, 3 and 8, respectively

The oocyte maturation and embryo development were performed in five replicates in all groups. Since the SCs are located in the testicular tissue at a lower temperature of the body temperature, it was presumed that these cells have the best performance at 33°C (Kastelic et al., 1996). Therefore, to mimic the body condition, the SCs were cultured at 33°C. On the other hand, since the oocyte maturation is carried out at 39°C and the co‐culture of SCs with oocyte at this temperature is inevitable, in another group the SCs were also cultured at 39°C, and eventually, the conditioned media were prepared at these temperatures. Generally, in this study, four followed groups were evaluated (illustrated in Figure 1):

1. Control group: including oocyte maturation in maturation medium.
2. Co‐culture group: including oocyte maturation in the maturation medium in co‐culture with the SCs
3. CM33 group: including oocyte maturation in the maturation medium with 10% CM prepared at 33°C
4. CM39 group: including oocyte maturation in the maturation medium with 10% CM prepared at 39°C

### 2.6 Evaluation of oocyte nuclear maturation

The nuclear status of oocytes was determined 22–24 h after the onset of oocyte maturation as previously described. In brief, the denuded oocytes were stained with 5 µg Hoechst 33342 in cooled ethanol for 5–10 min and then mounted into a small droplet of glycerol on a glass slide. Finally, the chromatin status of stained oocytes was examined and the oocytes were classified to germinal vesicle (GV; diffuse chromatin with a complete nuclear membrane), germinal vesicle breakdown (GVBD; slightly condensed or clumped chromatin with disappeared nuclear membrane), metaphase I (MI; condensed chromatin in the form of an irregular network of individual bivalents or a metaphase plate without polar body) or metaphase II (MI; the presence of a polar body or two shiny chromatin spots).

### 2.7 In vitro embryo production

The matured oocytes were exposed to high motile epididymal sperm capacitated in HEPES‐TCM containing 6 mg/ml bovine serum albumin (BSA) for 1 h and separated with swim‐up technique in HEPES‐SOF (synthetic oviductal fluid) containing 6 mg/ml BSA for 20 min. Oocytes were cultured in Tyrode's–albumin–lactate–pyruvate (TALP) medium supplemented with 25 µg/ml heparin and 6 mg/ml BSA and incubated with motile spermatozoa at 1 × 10^6^ spermatozoa/ml for 22–24 h at 39°C in an atmosphere of 9% CO2 in the air. After fertilisation, presumptive zygotes were mechanically denuded of their cumulus cells and cultured in synthetic oviductal fluid supplemented with amino acid and BSA (IVC‐SOFaaBSA) under mineral oil in 9% CO_2_ and 7% O_2_, with maximum humidified atmosphere. At this time, those oocytes with the pale and disintegrated cytoplasm were recorded as degenerated oocytes and then removed. The culture medium was refreshed on the third day of culture (day 0 defined as the day of fertilisation) with IVC‐SOFaaBSA containing 5% charcoal‐stripped FBS (CSS) and the culture was continued until 8 days post‐fertilisation.

### 2.8 Statistical analysis

The nuclear maturation status, oocyte fragmentation, cleavage, and blastocyst rates were analysed using one‐way ANOVA followed by Tukey's post hoc test. Statistical analysis was performed using IBM SPSS 25 software package. The results were reported as mean± SD and differences were considered significant at the level of *p* < 0.05.

## 3 RESULTS

**FIGURE 2:**
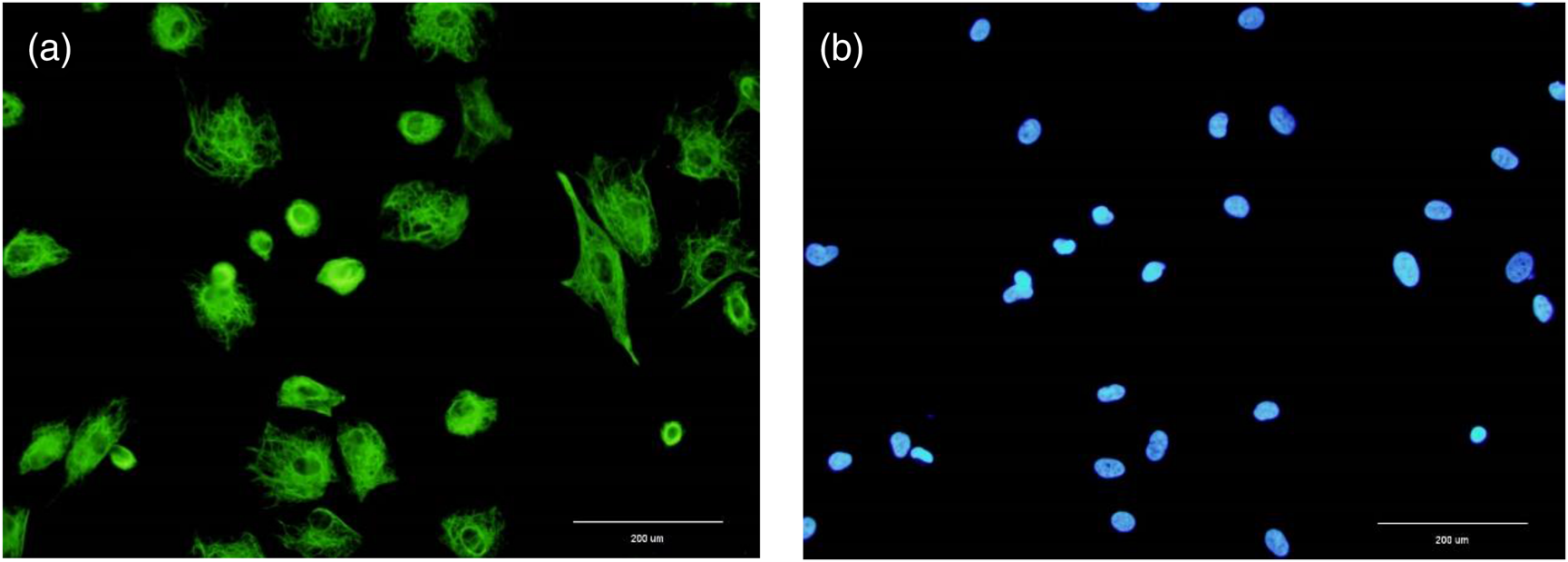
Immunocytochemical staining of ovine Sertoli cells using vimentin (a). Sertoli cell nuclei were identified using DAPI staining (b)

The presence of vimentin protein as a marker of SCs was confirmed by immunocytochemistry which showed more than 90% vimentin‐positive cells as SCs (Figure 2).

**FIGURE 3:**
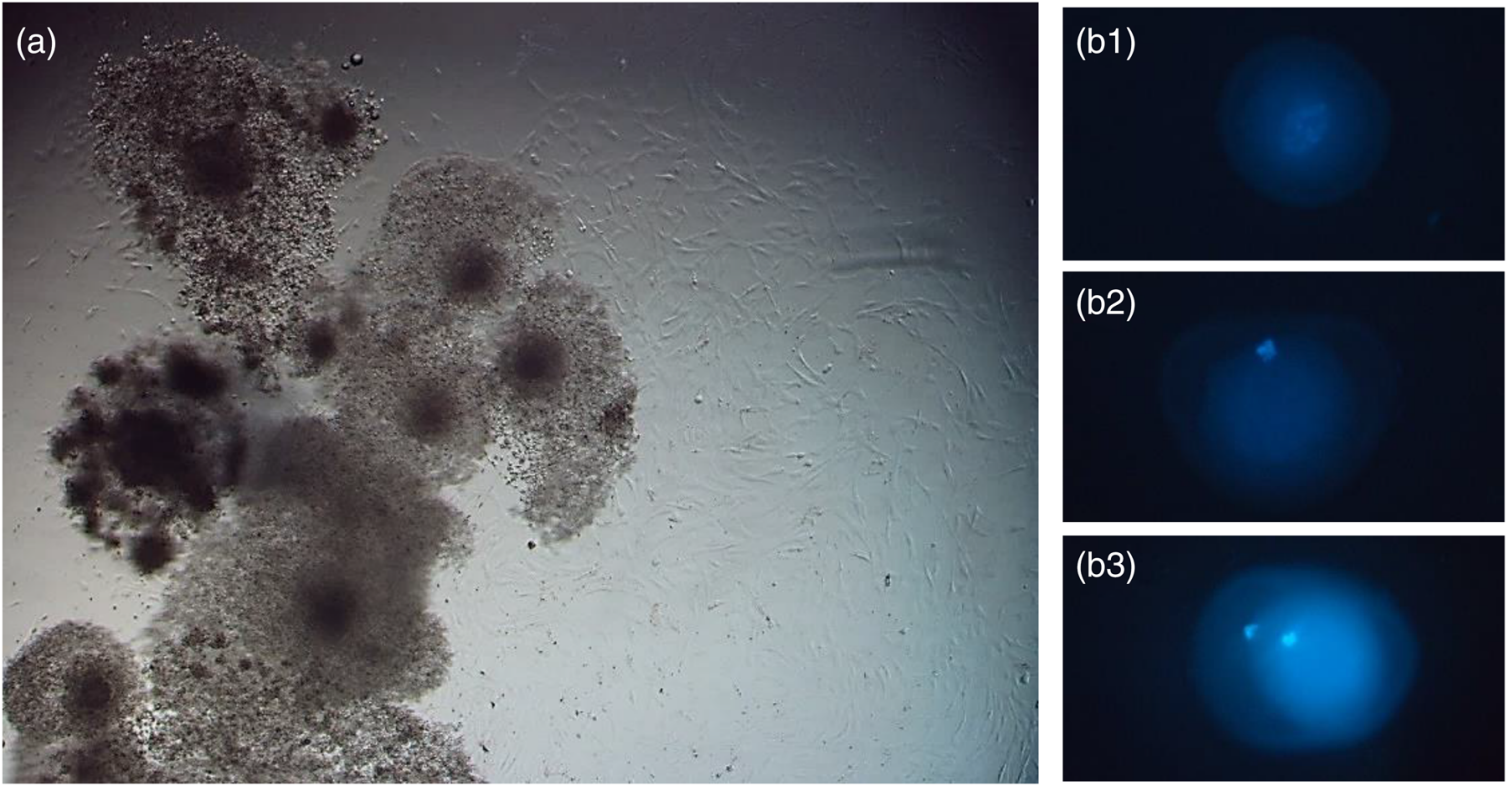
(a) Bovine oocytes co‐cultured with ovine Sertoli cells. (b) Nuclear staining of bovine oocytes after in vitro maturation. (b1) GVBD, (b2) M I, (b3) M II

To evaluation of oocyte maturation, the 318 bovine GV stage retrieved oocytes were cultured for 22–24 h in the absence (control group), presence of conditioned medium (CM33 and CM39) and in co‐culture with SCs (Figure 3). Significant differences were observed in meiosis resumption from GV to the MII stage in all treatment groups in comparison the control group (*p* < 0.05). However, there was no significant difference in IVM rates between treatment groups. Table 1 shows the number and percentage of oocytes in different nuclear maturation stages in all examined groups.

**TABLE 1:** The nuclear maturation of in vitro matured bovine oocytes in different groups

Groups	Oocyte No.	Nuclear status of in vitro matured oocytes (mean ± SD)			
		GV	GVBD	M I	M II
Control	85	0	1 (0.2 ± 0.45)	21 (24.34 ± 6.52)^a^	63 (75.66 ± 6.52)^a^
Co‐culture	85	0	0 (0 ± 0)	11 (12.69 ± 4.45)^b^	74 (87.31 ± 4.45)^b^
CM 33	76	0	0 (0 ± 0)	9 (10.95 ± 6.83)^b^	67 (89.05 ± 6.83)^b^
CM 39	72	0	0 (0 ± 0)	8 (11.40 ± 3.94)^b^	64 (88.60 ± 3.94)^b^

^a,b^ Different superscripts in the same column denote a significant difference (*p* < 0.05).

**FIGURE 4:**
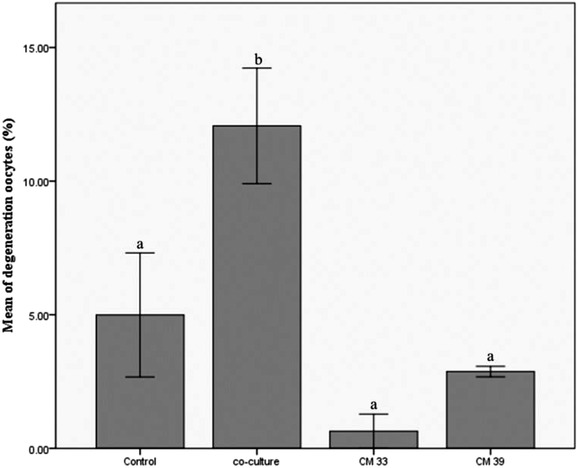
The degeneration rate of in vitro matured and fertilised bovine oocytes in different groups. The degeneration rate was determined 22–24 h after IVF, respectively. ^a,b^Different superscripts in the same column denote a significant difference (*p* < 0.05)

The number of degenerated oocytes was given in Figure 4. No significant difference was observed between the control group and the CM groups; but the degenerated oocytes in the co‐culture group (*n* = 16) were significantly higher than those in the control (*n* = 7), CM33 (*n* = 1) and CM39 (*n* = 4) groups (*p* < 0.05).

Table 2 shows the result of developmental competency of in vitro produced bovine embryos from different groups. The lowest cleavage rate was observed in the co‐culture group compared to the other groups (*p* < 0.05). But there was no significant difference between the other groups.

**TABLE 2:** The developmental rate of bovine embryos in different groups

Groups	Presumptive zygotes no.	Cleaved embryos no. (mean ± SD)	Blastocyst no. (mean ± SD)
Control	143	99 (69.28 ± 3.24)^a^	37 (25.50 ± 2.38)^a^
Co‐culture	135	80 (59.23 ± 3.45)^b^	29 (21.44 ± 2.63)^b^
CM 33	144	112 (77.89 ± 3.57)^a^	45 (31.38 ± 2.56)^c^
CM 39	141	109 (77.03 ± 4.57)^a^	35 (32.17 ± 4.60)^c^

^a,b,c^ Different superscripts in the same column denote a significant difference (*p* < 0.05).

The blastocyst rate was also lower in the co‐culture group than other groups (*p* < 0.05) and there was a significant difference between control and two CM groups (*p* < 0.05). No significant difference was observed between CM33 and CM39.

## 4 DISCUSSION

In the present study, for the first time, the effect of co‐culture of bovine oocytes with SCs on their maturation and the developmental competency of derived embryos were evaluated. Also, the conditioned medium of these cells was evaluated. The results showed that in all treatment groups, the number of MII oocytes was significantly higher than the control group. But the significantly higher degenerated oocytes and lower cleavage and blastocyst rates were observed in the co‐culture group.

As it has turned out, the success rate of development, implantation and pregnancy of IVM‐derived embryos are very low compared to the in vivo counterparts (Banwell & Thompson, 2008; Blondin et al., 2002; van de Leemput et al., 1999). Therefore, many studies attempted to use suitable factors to modify the maturation condition to give good embryo development with respect to rate and quality, closer to that observed in vivo in different species (Park et al., 2019; Richani et al., 2013; Sutton‐McDowall et al., 2012). It has been shown that co‐culturing oocyte with feeder cells has some beneficial effects on oocyte maturation and subsequent embryonic development (Lee et al., 2018), and it has been confirmed by most studies. In vitro maturation of dog oocytes for 48 and 72 h in co‐culture with embryonic fibroblasts increased in the percentage of M II oocytes (Hatoya et al., 2006). In this species, similar results were also achieved by oviduct epithelial cells co‐culture (No et al., 2018). Co‐culture of bovine COCs during IVM with Vero cells, enhanced their capability for cleavage and higher quality blastocyst production (Moulavi et al., 2006). In 2018, Lee et al. showed that co‐culture of porcine oocytes with human endothelial progenitor cells improves their maturation by regulating genes involved in oocyte maturation, cumulus cell expansion, and apoptosis and increases development of resulting embryos (Lee et al., 2018). In this regard, different other cells such as Vero cells in different species have been studied.

The SCs have been frequently used as a feeder layer in co‐culture with different cells such as neural stem cells (Shamekh et al., 2008), bone marrow‐derived mesenchymal stem cells (Zhang et al., 2016), endothelial cells (Fan et al., 2011) to increase their proliferation. One of the most widely used co‐cultures with SCs is the co‐culture of these cells with spermatogonia stem cells (SSCs), because of their in vivo compatibility with each other (Koruji et al., 2009). Co‐culture the SSCs with the SCs increases the number and diameter of colonies (Mohamadi et al., 2012; Pramod & Mitra, 2014), even more than adding some growth factors such as GDNF and SCF (Koruji et al., 2009). It should be noted that SCs have not been yet used in co‐culture with oocytes.

What was observed in the current study was the beneficial effect of a bovine oocyte co‐culturing with SCs on maturation rate, which was consistent with co‐culturing other cell types in previous studies; however, this beneficial effect in IVM was not observed in subsequent development of the resulting embryos. Presently, nothing is yet known about the mechanisms or factors that regulate the inhibiting and activating effects of the SCs on sperm in in vitro conditions. Nonetheless, to find reasonable answers to this negative effect we should consider all aspects of IVF in probable presence of SCs. The main issue is the presence of SCs attached to expanded cumulus cells of COCs, which inevitably during transferring the COCs to fertilisation medium. The presence of these cells may have had a negative effect on the process of sperm‐oocyte interaction. So, not only the number of degenerated oocytes increased in this group compared to other groups, but also showed a lower percentage of cleavage rate. The surface interaction has been observed in vivo between germ cells and SCs which the rate of adhesion decreases by the progression of differentiation and only partially retains by haploid germ cells (Ziparo et al., 1980); therefore, this adhesion may recur at the time of exposure and inhibits the sperm‐oocyte interaction; however, it needs further investigation. Interestingly, in CM groups, these negative effects were not observed and even CM improved the rate of development of the resulting embryos.

In recent years, the addition of cultured somatic cells‐CM in the culture medium during IVM or IVC has been considered. For instance, supplementation of maturation medium with 50% human bone marrow mesenchymal stromal cells‐CM significantly improved cytoplasmic and nuclear maturation of the GV stage of murine oocytes (Jafarzadeh et al., 2018). In addition, human umbilical cord stem cells‐CM improved oocyte maturation and mRNA expression related to apoptosis (Akbari et al., 2017). Lee et al showed potential efficacy of human adipose‐derived stem cells‐CM to reducing the ROS production in a culture medium by its active scavenging activity. They also demonstrated the effect of this CM on porcine oocyte development and the alteration of mRNA transcript levels in COCs (Lee, 2021). In another study, they suggested that human endothelial progenitor cell‐CM contains several essential growth factors for porcine oocyte and subsequent embryo developments (Lee, 2020). The supportive effect of testicular cell conditioned medium on IVM of mice oocytes and their morphology was also determined by Adib et al. (2020). Our findings were consistent with the results of these studies. The results indicated SC‐CM provides good or better support than the cells themselves for developmental competency of embryos derived from treated oocytes, which would be more appealing to scientists for oocyte development as SC‐CM is a non‐cellular liquid and excludes the potential possibility of contamination, more convenient than co‐culture and does not have the disadvantages of inevitable attaching SCs to COCs. Besides, it can be assumed that SCs also consumed the nutrients and growth factors in culture medium during IVM to maintain their physiological characteristic and function.

Another important point studied in this study was the optimum temperature at which SCs were expected to have the best function. It has been shown that the intratesticular temperature in live ram is about 33°C (Kastelic et al., 1996). However, due to this fact that the IVM is performed in ovine body temperature (39°C), co‐culture with SCs at this temperature was inevitable. Therefore, to investigate the effect of temperature on Sertoli cell function, the CM was prepared at 33°C and 39°C and used in IVM. The results of this study showed that SCs culture temperature had no effect on their performance.

## 5 CONCLUSION

It can be concluded that the Sertoli cells can be suitable for co‐culturing with oocytes during IVM, but detrimental for subsequent embryo development. In turn, Sertoli cells‐derived conditioned medium (SC‐CM) can provide sufficient bioactive materials with COCs for enhancing oocyte competence and embryo development.

## AUTHOR CONTRIBUTIONS

In this study Sina Nazifi contributed to the planning and performance of the study; Hassan Nazari contributed to the planning and performance of the study, and writing and revising of the manuscript; Hossein Hassanpour contributed to the planning of the study, and revising the manuscript; Ebrahim Ahmadi contributed to the planning the study and statistical analysis; Azita Afzali contributed to performance of the study.

## CONFLICT OF INTEREST

None of the authors have any conflict of interest to declare.

### ETHICS STATEMENT

Animal husbandry and handling were conducted in accordance with the guidelines of the ethical committee of Shahrekord University, Iran.

### PEER REVIEW

Yes, I would like my name to appear with my report on Publons at https://publons.com/publon/10.1002/vms3.939.

## Data Availability

The data that support the findings of this study are available from the corresponding author upon reasonable request.
